# Tocilizumab Induces IL-10-Mediated Immune Tolerance in Invasive Candidiasis

**DOI:** 10.3390/jof7080656

**Published:** 2021-08-13

**Authors:** Zhaohong Tan, Michelle Meng Huang Mok, Win Mar Soe, Thomas Paulraj Thamboo, Jessamine Geraldine Goh, Qi Hui Sam, Motomi Osato, Sharada Ravikumar, Louis Yi Ann Chai

**Affiliations:** 1 Department of Medicine, Division of Infectious Diseases, National University Health System, Singapore 119228, Singapore; aiden.tan@hotmail.com (Z.T.); winmarsoe18@gmail.com (W.M.S.); jessamine_goh@sris.a-star.edu.sg (J.G.G.); qihui.sam@nus.edu.sg (Q.H.S.); sharada_ravikumar@nuhs.edu.sg (S.R.); 2 Cancer Science Institute of Singapore, National University of Singapore, Singapore 117599, Singapore; micmok@nus.edu.sg (M.M.H.M.); csimo@nus.edu.sg (M.O.); 3 Department of Pathology, National University of Singapore, Singapore 117596, Singapore; pattpt@nus.edu.sg; 4 National University Cancer Institute, National University Health System, Singapore 119074, Singapore; 5 Yong Loo Lin School of Medicine, National University of Singapore, Singapore 119228, Singapore

**Keywords:** *Candida albicans*, interleukin 6 blockade, inflammation, immunomodulation, monoclonal antibody

## Abstract

The existence of a hyperinflammatory state has been observed in patients with invasive fungal infections (IFI). It is being postulated whether morbidity from IFI may, in part, be a consequence of an unnecessarily prolonged or exaggerated proinflammatory immune response including interleukin 6 (IL-6) post-infection, in a host with dysregulated or compromised immunity. This, in turn, induces collateral host injury at the tissue and organ level, leading to adverse outcomes. Tocilizumab has become widely used as an immunomodulator in the treatment of inflammatory conditions. Here, we evaluated the use of tocilizumab to curb post-infective inflammatory flare in the setting of an in-vivo mouse model for invasive candidiasis. Following *Candida* infection, the tocilizumab-treated mice showed improved short-term survival compared with the saline-treated control mice. There was a reduced inflammatory response mounted by the host, coupled with reduced IL-6 but increased IL-10 levels. TNF-α and IFN-γ responses were not affected. Tocilizumab facilitated immune tolerance by selectively inducing IL-10, producing CD8α+ conventional dendritic cells (DCs) and peripheral T-regulatory cells, over CD11b+ conventional DCs and plasmacytoid DCs. We demonstrate here the sequelae from immunomodulatory manipulation and the basis whereby the use of monoclonal antibodies may be further explored in IFI.

## 1. Introduction

Morbidity and mortality rates have remained high in invasive fungal infections (IFI), of which Candidemia is the fourth most common cause of hospital-acquired bloodstream infection worldwide [1]. Despite the current array of anti-fungal armamentarium available for treatment, all-cause mortality related to invasive candidiasis is still in excess of 30%. These figures are especially significant given the ever-increasing numbers of immunocompromised patients who are at-risk in the specialties of hematology, oncology, transplantation, and critical care medicine [2].

It is well-recognized that susceptibility to IFI and adverse outcomes are attributable to an impaired host immune response, which is inadequate at mounting an adequate defense upon encountering the fungal pathogen. These circumstances are typically relatable in the context of immunosuppressed haemato-oncology and transplant patients. Conversely, it has been postulated whether morbidity from IFI may, in part, be a consequence of an unnecessarily prolonged or exaggerated proinflammatory immune response post-infection, which, in turn, induces collateral host injury at the tissue and organ level [3,4]. The existence of elevated inflammatory immune states following IFI have been observed, for instance, in patients with invasive aspergillosis, whereby persistently elevated interleukin 6 (IL-6) levels were predictive of eventual adverse outcomes [5]. A state of immune reconstitution inflammatory syndrome existent in fungal infections is progressively being recognized [6,7] as a rationale to explain the unanticipated clinical deterioration at times after the initiation of anti-fungal treatment. Consequently, this raises the question whether the modulation of an inflammatory flare following fungal infection may in turn alter outcomes.

Tocilizumab is an anti-interleukin 6 receptor antibody that, in the past decade, has become widely used as an immunomodulator in the treatment of autoimmune and inflammatory conditions [8,9]. Here, we aim to study the utility of tocilizumab to quench the inflammatory flare following fungal infection in the setting of an in-vivo mice model for invasive candidiasis.

## 2. Materials and Methods

### 2.1. Preparation of Stimuli

Preparation of *C. albicans* was done using a previously well characterized *C. albicans* clinical strain (SC 5314). *C. albicans* was grown in Sabouraud Dextrose Liquid Medium (Thermo Fisher Scientific, Waltham, MA, USA) for 16 h at 37 °C. *C. albicans* blastospores were isolated by centrifugation of the suspension at 4000 rpm for 10 min, discarding the media and re-suspending the blastospores in PBS. Unwanted hyphae were removed by passing the suspension through sterile gauze. For heat killed *C. albicans,* the blastospores were centrifuged, and the pellets were washed twice in sterile PBS and resuspended to a density of 4 × 10^8^ cells/mL before boiling at 100 °C for 60 min. Toll like receptor (TLR) 4 ligand, lipopolysaccharide (LPS) (from *Escherichia coli* K12 strain, InvivoGen, San Diego, CA, USA) was prepared according to the manufacturer’s instruction. The TLR 2 ligand, Pam3Cys (P3C) (EMC Microcollections, Tuebingen, Germany), was reconstituted in endotoxin-free water (Gibco, Waltham, MA, USA).

### 2.2. Disseminated Candidiasis Mice Model

All of the animals were housed in the animal facility at the Biological Resource Centre, Singapore. The animals were handled following Singapore’s “Guidelines on the Care and Use of Laboratory Animals for Scientific Purposes”. Experiments were conducted after approval by the Institutional Animal Care and Use Committee (IACUC) under protocol 181308 (12 September 2018).

### 2.3. Infection

**Figure 1:**
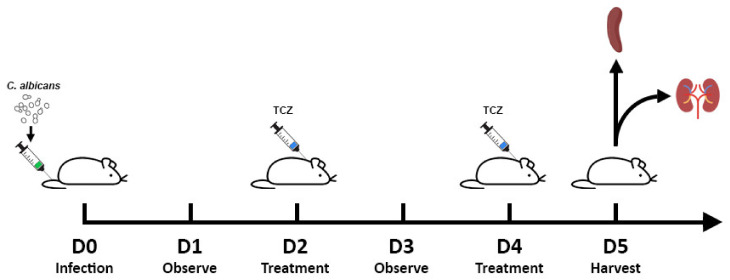
Disseminated candidiasis mice model. Schematic diagram of administration of *C. albicans* and tocilizumab. Mice were infected with *C. albicans* on D0, followed by treatment with either tocilizumab or saline on D2 and D4, and the study was terminated on D5. Spleens and kidneys were removed. Spleens were crushed and splenocytes were obtained for stimulation with the respective ligands. One set of the pair of kidneys were used for histological studies and the other for fungal load quantification.

Seven- to eight-week-old wild type male BALB/c mice were obtained from InVivos, Singapore. On Day 0 (D0), 100 µL of *C. albicans* blastospores (2 × 10^6^ CFU/mL; 5 × 10^6^ CFU/mL for survival study) suspended in PBS was injected into the tail veins of the mice. The mice were observed on D1. On D2, 100 µL of tocilizumab (5 mg/mL; Genentech, CA, USA) was injected into the nape of the treatment group mice, while 100 µL of saline was injected into the nape of the control group mice. The mice were then observed again on D3 and tocilizumab or saline treatment was repeated on D4. On D5, the mice were sacrificed. The kidneys were harvested for fungal load quantification and histological studies, and the spleens were harvested for in vitro splenocyte stimulation and flow cytometry. The timeline of infection and treatment is as depicted in Figure 1.

### 2.4. Fungal Load Quantification

The kidneys were removed from each mouse and were homogenized and diluted in serial 10-fold dilutions. These were plated on Sabouraud Glucose Agar with Chloramphenicol (Sigma-Aldrich, St. Louis, MO, USA) and incubated at 35 °C. After 24 h, the fungal colony forming units (CFUs) on the agar plate were enumerated.

### 2.5. Histology

The kidneys were removed from each mouse and placed in 10% formalin and were subsequently processed to paraffin blocks. Sections of the kidney tissue were cut at 4 µm and stained with haematoxylin and eosin (H&E) to visualize the host response, and Gomori methenamine silver (GMS) stain to identify the fungal structures. Slides were viewed on an Olympus BX41 microscope and representative images were captured at ×100 and ×200 original magnification using an Olympus DP22 digital camera attached to the microscope.

### 2.6. Stimulation and Cytokine Measurement

Splenocytes were obtained from the mice on D5 of infection by crushing the spleens. Then, 5 × 10^5^ cells were added to each well in a 96-well plate. The cells were stimulated in duplicate with heat-killed *C. albicans* (CAN; 1 × 10^6^ organisms/mL), LPS (10 ng/mL), P3C (10 µg/mL), or in RPMI. After incubation at 37 °C for 24 or 48 h, supernatant was collected, and cytokine measurements were made using enzyme-linked immunosorbent assay (ELISA).

Interferon (IFN)-γ, interleukin (IL)-6, IL-10, and tumor necrosis factor alpha (TNF-α) release in the supernatants, representative of the inflammatory and regulatory arms of host immunity, were measured using commercially available ELISA kits (eBioscience, San Diego, CA, USA) according to manufacturers’ instructions. The lowest detection limits were as follows: IFN-γ—16 pg/mL, IL-6—4 pg/mL, IL-10—32 pg/mL, and TNF-α—8 pg/mL.

### 2.7. Statistics

In vitro experiments were performed in duplicates. The results were pooled from at least three sets of separate experiments. In vivo experiments were performed using at least five mice in each group. The results were pooled from at least three sets of mice experiments. The results were analyzed using GraphPad Prism (v7, San Diego, CA, USA). Log-rank test and Mann–Whitney U test were used for the statistical analysis. The level of significance was set at *p* <0.05.

### 2.8. Flow Cytometry

The splenocytes and blood were harvested from mice. The RBCs were lysed with an ACK lysis buffer and the splenocytes were washed once in cold 1× PBS. The cells were then stained using PerCP/Cyanine5.5 CD19, PE/Dazzle 594 I-A/I-E, APC/Cyanine7 B220, Brilliant Violet 421 CD11c, APC CD11b, BV510 CD8a, and FITC Ly-6G/Ly-6C (Gr-1) anti-mouse antibodies (Biolegend, San Diego, CA, USA) to differentiate between conventional dendritic cells (cDCs) and plasmacytoid DCs (pDCs). Alexa Fluor 643 FOXP3, PE/Cyanine7 CD4, and FITC CD25 anti-mouse antibodies were used to differentiate T-regulatory (Treg) cells in peripheral blood. PE IL-10 anti-mouse antibody was used for intracellular staining of the DCs and Treg cells. Stained cells were analyzed on the Becton–Dickinson LSRII Flow Cytometer (BD Biosciences, Franklin Lakes, NJ, USA). Data analysis were performed using FlowJo software (v7.6.5; Tree Star, Ashland, OR, USA).

## 3. Results

### 3.1. Tocilizumab-Treated Mice Showed Lower Short-Term Mortality despite No Reduction in Fungal Burden

**Figure 2:**
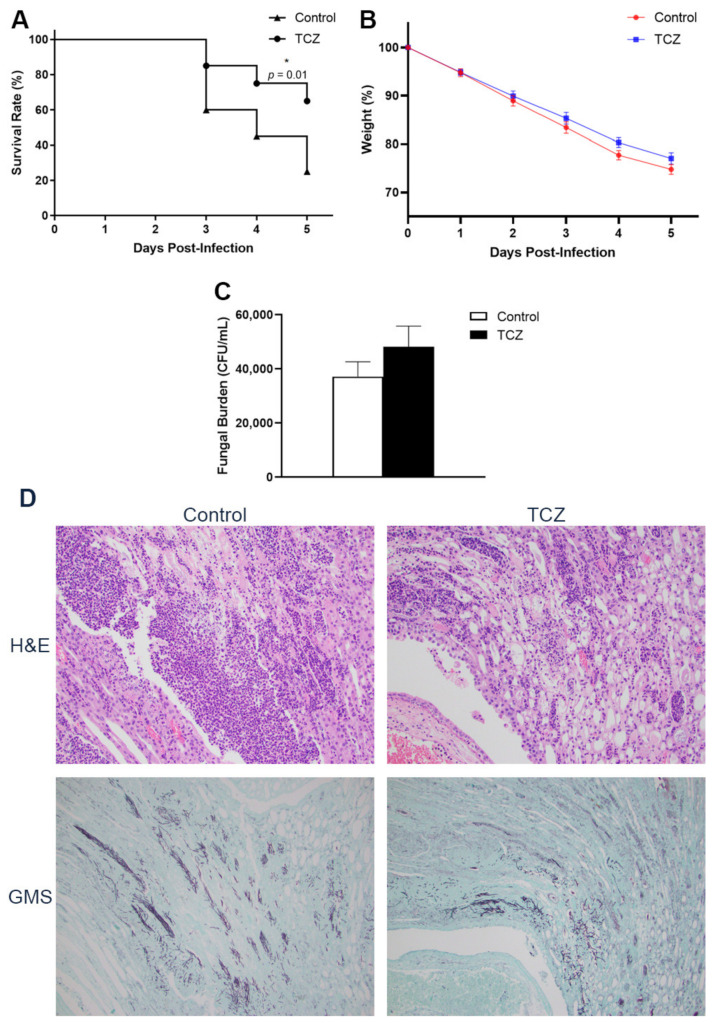
Survival rate, weight, and fungal burden of infected mice treated with tocilizumab (TCZ). Mice were infected with *C. albicans* on D0 followed by treatment with either saline or TCZ on D2 and D4, and the study was terminated on D5. The weight and survival rate were recorded from D0 until the termination of study at D5. One set of the pair of kidneys were used for histological studies and the other for fungal load quantification. (**A**) Survival rate of control vs. TCZ-treated mice and *n* = 20, pooled from three experiments * *p* < 0.05 using log-rank test. (**B**) Weight and (**C**) fungal load of control vs. TCZ-treated infected mice and *n* = 25, pooled from four experiments (mean ± SEM). (**D**) Kidneys from control and TCZ-treated groups were stained with H&E (top panel; 200× magnification) to visualize the host response and GMS (bottom panel; 100× magnification) to identify the fungal structures.

Following infection by *C. albicans*, the mice that were treated with tocilizumab showed better short-term survival than the mice given saline as the control (Figure 2A). Between the tocilizumab-treated and control mice, there was no difference in the weight loss (Figure 2B). Comparing the fungal burden in the kidneys after infection, there were no statistically significant differences between tocilizumab-treated and control groups, although the tocilizumab-treated mice showed a trend towards slightly higher fungal colony counts (Figure 2C). Histologically, there was less marked inflammatory response in the infected kidneys of treated mice compared with the controls. Here, differences in fungal burden were not apparent between the two groups of mice (Figure 2D).

### 3.2. Tocilizumab Treatment Results in Elevated Interleukin-10 Response

**Figure 3:**
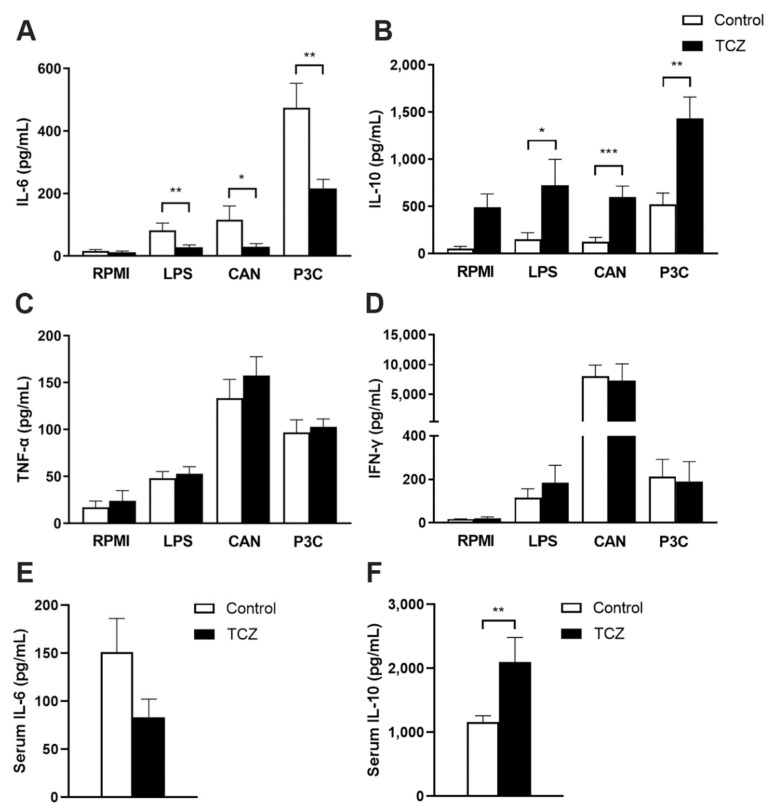
Mice were infected with *C. albicans* on D0 followed by the treatment with either saline or tocilizumab (TCZ) on D2 and D4, and the study was terminated on D5. The spleens were obtained and crushed, and the splenocytes were stimulated with RPMI, LPS, CAN, and P3C. Supernatants from splenocyte stimulation were isolated and (**A**) IL-6, (**B**) IL-10, (**C**) TNF-α, and (**D**) IFN-γ levels were quantified by ELISA and *n* = 20, pooled from five experiments (mean ± SEM). (**E**) Serum IL-6 and (**F**) serum IL-10 quantified by ELISA in the control vs. TCZ-treated mice and *n* = 10, pooled from two experiments (Mean ± SEM). * *p* < 0.05, ** *p* < 0.01, *** *p* < 0.001 for splenocytes or sera cytokines from the control vs. TCZ-treated mice using Mann–Whitney U test.

Tocilizumab treatment significantly reduced IL-6 production in mice splenocytes that were stimulated with *Candida*, as well as with LPS and P3C through TLR4 and TLR2 pathways, respectively (Figure 3A). The effects of tocilizumab treatment on amplifying the IL-10 response were clearly seen when splenocytes were stimulated with *Candida,* LPS and P3C as ligands (Figure 3B). On the other hand, TNF-α and IFN-γ production were not affected by tocilizumab (Figure 3C,D). The circulating cytokines in the sera of the mice showed similar trends: IL-10 was significantly elevated in tocilizumab-treated mice with conversely reduced IL-6 (Figure 3E,F).

### 3.3. Tocilizumab Induced IL-10-Secreting CD8α+ Dendritic Cells and Peripheral T Regulatory Cells

**Figure 4:**
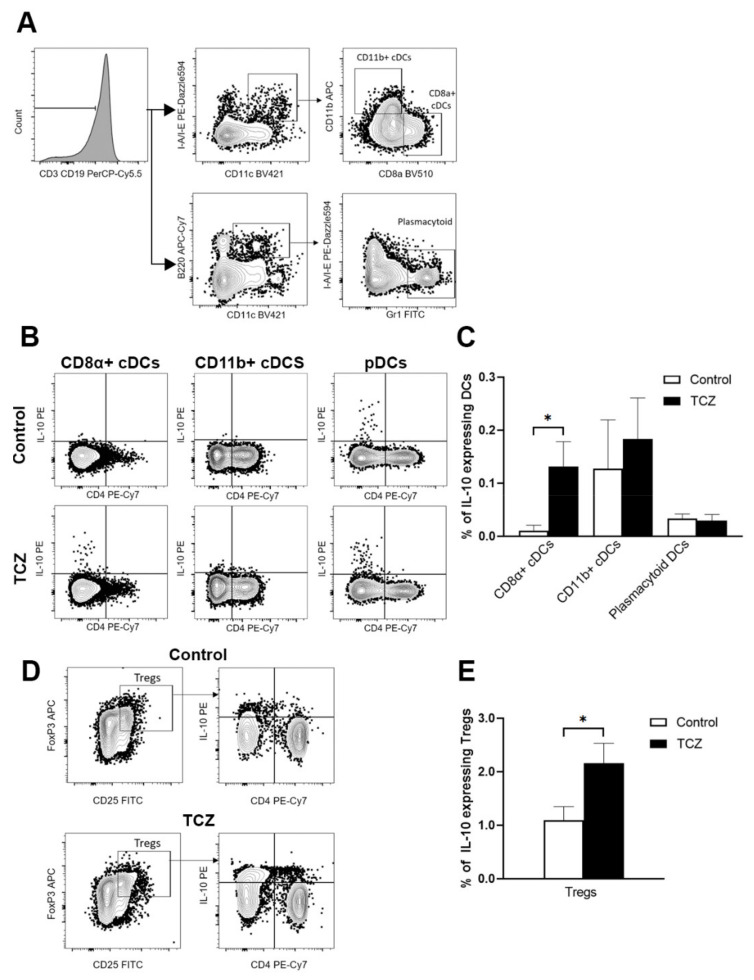
FACS gating strategy and percentage population. Mice were infected with *C. albicans* on D0, followed by treatment with either tocilizumab or saline on D2 and D4. Splenocytes were harvested on D5, and enumerated and stained with fluorescence-conjugated antibodies to be analyzed by FACS. (**A**) Gating strategy used to sort DCs into conventional CD8α^+^, CD11b+, and plasmacytoid DCs. (**B**) Representative FACS plots of IL-10 expressing DC populations in control vs. treated groups. (**C**) Percentage DC population of IL-10 producing DCs in splenocytes and *n* ≥ 5. * *p* < 0.05 for cell populations of control mice vs. cell populations of TCZ-treated mice using Mann–Whitney U test (mean ± SEM). (**D**) Representative FACS plots of peripheral blood cells being sorted into IL-10 expressing regulatory T cells. (**E**) Percentage population of IL-10 producing regulatory T cells (Tregs) in peripheral blood (mean ± SEM). * *p* < 0.05 for cell populations of treated mice vs. control mice using the Mann–Whitney U test.

Following on the above findings, we queried if tocilizumab treatment could have induced a tolerogenic state in the host. We looked at dendritic cells (DCs) given their role in bridging the innate immune function and in maintaining tolerance (Figure 4). We saw a clear induction of IL-10 secreting CD8α+ conventional DCs (cDCs) by tocilizumab over CD11b+ cDCs and plasmacytoid DCs in the spleens of the treated mice (Figure 4B,C). Correspondingly, there was increased IL-10-expressing T regulatory cells in the peripheral blood of the treated group (Figure 4D,E).

## 4. Discussion

A central aim of this study was to investigate the basis and outcome of modulating the inflammatory response observed in invasive fungal infection. Here, we utilized an in-vivo candidiasis mice model and employed tocilizumab to curb early IL-6 flare, which has been observed by some [5,10]. Interleukin 6 is a pleiotropic cytokine responsible for the induction of acute phase proteins, T & B cell differentiation [11], and facilitation of type 17 helper T cell (Th17) response anticipated in IFI [12,13]. Dysregulated IL-6 may exert a pathologic effect through overt inflammation. On this basis, anti-IL-6 monoclonal antibodies are now used as treatment in a range of rheumatological and immune-mediated diseases. The restrained immunosuppressive potency of tocilizumab and its relative safety profile [14] favored its use here, whereby the intent was to minimize overtly subverting the host immune response at the onset of infection.

The experiments were carried out without anti-fungal treatment to elucidate the effects of IL-6 modulation as per the intent of this study. We have shown here, notably, that tocilizumab treatment following *Candida* infection led to improved short-term survival. Overall, there were no outright differences in fungal burden between the groups receiving tocilizumab and the controls. However, mindful of its immunomodulatory effects, we could not help noticing the tendency towards a slight increased fungal burden coupled with reduced inflammatory response in the tocilizumab-treated mice; there was no difference in weight loss between the treated and control mice.

The improved short-term survival despite a possible converse trend in colony count led us to speculate whether tocilizumab induced a state of immune tolerance. This hypothesis was first supported by the clear induction of IL-10 in the sera and following *Candida,* LPS and P3C stimulation in the splenocytes of the mice that received tocilizumab treatment. The effects of tocilizumab were delineated as TNF-α and IFN-γ production were not affected. Incidentally, there have been observations that tocilizumab exerted limited inhibition on IL-6 production as seen in mice cells [15] and in DCs [16], indicating that tocilizumab might exert its activity through alternative non-IL-6 pathways.

Dendritic cells orchestrate bridging of the innate and acquired arms of the host immune response. Tissue level resident cDCs, broadly split into CD8α+ and CD8α-CD11b+ subgroups, are involved in immune sensing and antigen presentation. Here, we found that one of the ways that tocilizumab induced tolerance was through CD8α+ cDCs, with a noticeably increased IL-10 expression. CD8α+ cDCs are known to be well placed in marginal zones to filter blood antigens [17], and then migrating to the T cell zones of the spleen to interact with CD8 T cells to facilitate a state of tolerance [18,19]. An augmented IL-10 environment facilitates an immune tolerogenic milieu. This, together with the demonstrated increased co-expression of IL-10 by the CD8α+ cDCs, is in line with DCs performing a regulatory role induced through tocilizumab. In turn, we also showed the increased regulatory T cells peripherally. CD8α+ DCs are recognized to be distinct and most efficient in their capability for cross presentation, unique from non-CD8+ DCs [20] permitting antigen presentation for induction of tolerance [21]. IL-6 blockade leading to increased Treg and immune tolerance [22] or through M2 macrophages have been described [23]. We demonstrate here a novel aspect of peripheral tolerance through CD8α+IL10+ cDCs triggered by tocilizumab.

A perceivable concern from immunomodulation by tocilizumab is the facilitation of a state of tolerant co-existence of the fungal pathogen within the host tissue, and this was in line with our experimental observations on the fungal burden. While the curtailment of the inflammatory response does appear to be of some benefit, at least transiently to the host, the persistence of the fungi will remain a concern, especially after the effect of tolerance wears off. In the setting of chronic infections such as malaria and Hepatitis C, stimulation of T regulatory cells has also been observed [24,25,26]. However, the benefits or risks of attempting to manipulate this regulatory arm of the immune system for therapeutics remain unknown.

The limitation of our experimental setup did not permit extrapolating any findings on the effects of tocilizumab beyond the acute phase of *Candida* infection. It is to be highlighted that the intention of this study is not to promulgate tocilizumab for consideration as the sole treatment modality for IFI. Rather, it is to investigate the underlying mechanism and how immunomodulation may possibly alter the outcome in IFI, especially in the current realm of interest for their use in infections with recognized cytokine or inflammatory flares. The recent times have witnessed expanded use of immunomodulators including tocilizumab to complement treatment of infection-related inflammatory conditions with variable results [27,28]. Questions arising on the extent of immune suppression, variability of dosing and timing of immunomodulation relative to infection, and the pertinence to the human host remain to be further explored. Immunomodulation can be a double-edged sword. In the context of invasive candidiasis, tocilizumab treatment tends to confer a state of immune tolerance, though possibly at the expense of persistence of the fungi in the host.

## Data Availability

Data sharing not applicable.
